# Use of a total traffic count metric to investigate the impact of roadways on asthma severity: a case-control study

**DOI:** 10.1186/1476-069X-10-52

**Published:** 2011-06-02

**Authors:** Angus G Cook, Annemarie JBM deVos, Gavin Pereira, Andrew Jardine, Philip Weinstein

**Affiliations:** 1 School of Population Health, University of Western Australia, Perth, Australia; 2 CRC Asthma and Airways, Sydney, Australia; 3 Institute of Health Policy & Management, Erasmus University, Rotterdam, Netherlands; 4 Queensland Health, Brisbane, Australia; 5 University of South Australia, Adelaide, Australia

## Abstract

**Background:**

This study had two principal objectives: (i) to investigate the relationship between asthma severity and proximity to major roadways in Perth, Western Australia; (ii) to demonstrate a more accurate method of exposure assessment for traffic pollutants using an innovative GIS-based measure that fully integrates all traffic densities around subject residences.

**Methods:**

We conducted a spatial case-control study, in which 'cases' were defined as individuals aged under 19 years of age with more severe asthma (defined here as two or more emergency department contacts with asthma in a defined 5-year period) versus age- and gender-matched 'controls' with less severe asthma (defined here as one emergency department contact for asthma). Traffic exposures were measured using a GIS-based approach to determine the lengths of the roads falling within a buffer area, and then multiplying them by their respective traffic counts.

**Results:**

We examined the spatial relationship between emergency department contacts for asthma at three different buffer sizes: 50 metres, 100 metres and 150 metres. No effect was noted for the 50 metre buffer (OR = 1.07; 95% CI: 0.91-1.26), but elevated odds ratios were observed with for crude (unadjusted) estimates OR = 1.21 (95% CI: 1.00-1.46) for 100 metre buffers and OR = 1.25 (95% CI: 1.02-1.54) for 150 metre buffers. For adjusted risk estimates, only the 150 metre buffer yielded a statistically significant finding (OR = 1.24; 95% CI:1.00-1.52).

**Conclusions:**

Our study revealed a significant 24% increase in the risk of experiencing multiple emergency department contacts for asthma for every log-unit of traffic exposure. This study provides support for the hypothesis that traffic related air pollution increases the frequency of health service contacts for asthma. This study used advanced GIS techniques to establish traffic-weighted buffer zones around the geocoded residential location of subjects to provide an accurate assessment of exposure to traffic emissions, thereby providing a quantification of the ranges over which pollutants may exert a health effect.

## Background

### Asthma and traffic emissions

Traffic emissions contain a complex mixture of particulate matter (PM), oxides of nitrogen (NOx), carbon monoxide (CO), oxides of sulphur (SOx), unburned hydrocarbons, and other volatile organic compounds (VOCs) [1]. There is increasing evidence that residential proximity to major roadways is associated with an elevated risk of asthma exacerbations and greater severity of symptoms [2-6]. A number of epidemiological analyses of the localised impact of traffic emissions on respiratory health have demonstrated associations with airway inflammation and lung function changes in the general population, and these effects are more detrimental to asthmatics because of their already compromised pulmonary function [7,8]. Two recent reviews have concluded that there was a consistent association between asthma and reduced lung function and living near highly trafficked roads [9,10].

### Exposure metrics for traffic emissions

The complex combination of chemicals present in traffic emissions, and their correspondingly variable dispersal with distance, has contributed to the lack of consensus on the distance at which their impact diminishes to "safe" levels [11,12]. Various measures of exposure to traffic have been evaluated, ranging from distances to roadway to the use of distance-weighted traffic counts [13-17]. Distance to major roadway is one of the more commonly used metrics which assumes that the majority of an individual's exposure is likely to derive from the nearest major road to which their house is located. Holguin demonstrated that road density - a proxy measure for exposure - within the 50- and 100-metre buffers around homes was associated with reduced lung function and increased exhaled NO in children with asthma [12].

Geographic Information Systems (GIS) have been used in previous studies to map the residential locations of individuals with asthma by postcode [18] or by the exact longitude or latitude coordinates of the residential address. This geocoding of addresses has enabled researchers to determine the exact residential proximity of individuals to the nearest major road [15,19]. For example, in a 1996 analysis of asthma presentations at general practices in London, Livingstone [20] used GIS to estimate the shortest distance of residential postcode to a busy road. No increase in risk of general practitioner diagnosis of asthma was identified in those living close to busy roads.

A limitation of these methods is that they fail to account for the exposure received from other roads surrounding the residential location. Few studies have used the more accurate exposure model which determines the exact traffic flow within a circular buffer surrounding the residential address of cases. A case-control study conducted by Modig [21] used a GIS-generated 200 m radius buffer around each case and control and aggregated mean traffic flow (vehicles per 24 hour period, weekday) of all roads contained within the buffer zone. In the case-control study by Wilkinson [22], centroids of the residential postcodes were used to determine the co-ordinates of the residential locations. In addition to residential proximity to a major road, traffic volume was also computed and used as an additional measure of exposure. However, no association was found between risk of hospital admission for asthma or respiratory illness and proxies for traffic related pollution.

### Measuring asthma severity

Over the past few decades, the prevalence of asthma has steadily increased. Australia has one of the highest prevalence rates of asthma in the world [23] and a 2005 report estimated that costs associated with asthma comprised 1.4% of the national total health expenditure [24]. There is evidence that rates of asthma may be plateauing in developed countries but continuing to climb in the developing world [25]. There remains ongoing controversy over whether the severity of asthma is increasing [26]. Asthma severity may be measured in various ways, including the frequency and degree of respiratory symptoms, changes in lung function (usually measured in relation to flow and volume) and degree of utilisation of health services, such as general practitioners and hospitals. Patterns of medication usage have also been evaluated, and those used for major asthma attacks - such as nebulisers and oral steroids - appear to be valid markers for asthma severity [27]. It has been noted that many of these indicators also reflect issues of asthma control and management, as opposed to simply the "underlying" degree of clinico-pathology [28]. Increasing hospital admission rates have previously been observed in the UK and New Zealand, and this seems to reflect true changes in disease severity as opposed to trends in admission procedures[27].

One of the potentially important factors driving increased asthma severity is exposure to outdoor air pollutants, including traffic emissions. This study sought to achieve two major objectives: (i) to investigate the relationship between asthma severity and proximity to major roadways in Perth, Western Australia; (ii) to demonstrate a more accurate method of exposure assessment for traffic pollutants using an innovative GIS-based measure that fully integrates all traffic densities around subject residences. Unlike past studies, our analysis provided a consistent system of weighting for the mean traffic flow for each road, taking into account traffic flows and the lengths of each road contained within the area of exposure.

## Methods

### Study Design

This study is a spatial case-control study using geocoded emergency department (ED) presentation data (2002-2006) for children and young adults, aged 0-19 years, in the city of Perth, Western Australia.

### Study Area

The study area in south-western region of the Perth metropolitan area was chosen because it is traversed by a combination of major metropolitan vehicle corridors and less trafficked local roads, thereby providing reasonable degree of exposure contrast. These corridors are uniformly at the top of Perth's list of "priority action locations", which are defined by the Department of the Environment based on 13 road network assessment criteria, including traffic congestion and vulnerability to very high levels of motor vehicle exhaust emissions [29]. The total population in the area was 269,734 (2006 Census of Population and Housing). Overall, Perth is a city with relatively low levels of traffic-related air pollution, and a limited contribution of industrial air pollution relative to that arising from motor vehicle traffic.

The study area included 613 census Collection Districts (CDs), encompassing eight Statistical Local Areas. CDs are the smallest available geographical areas for which demographic statistics are disseminated by the Australian Bureau of Statistics, and on average include 225 dwellings.

### Study population

Subjects were individuals aged less than 19 years with residential addresses in the study area, who presented at a public emergency department (ED) of any Perth metropolitan hospital between 2002-2006 with a principal diagnosis of asthma (ICD-10 code J45) or status asthmaticus (J46). The hospitals chosen for the study provide the major avenue for emergency care in metropolitan Perth.

### Case and control definitions

Given that the primary focus of this analysis is on severity, "cases" were defined as those with more than one ED contact for asthma in the defined period, whereas "controls" were defined as those with only one emergency department contact in the same time period. This approach is comparable to other case-control studies of asthmatics based on other severity indicators, such as those in which cases and controls are defined by different levels of medication use [30].

This selection procedure contrasts with asthma studies that use controls who are "asthma-free" for comparison. In such studies, selection of the control population is often defined by some other non-respiratory condition that also warrants medical attention (such as gastroenteritis). This form of case and control specification is used to assess the likelihood of presence or absence of asthma, as opposed to levels of severity of pre-existing disease as in this study.

### Matching procedure

Each case was individually-matched to one control of the same gender and 5-year age category (0-4, 5-9, 10-14, 15-19 years). The season in which the ED contact was also partially matched for both cases and controls, such that at least one of the case's ED contacts occurred in the same season as the control's single recorded contact.

### Retrieval of subject Information

De-identified data for the period 2002-2006 were obtained from the Emergency Department Information System (EDIS) from all the major public hospitals in Perth. The system draws on real time, continuously updated information on ED presentations, including the coded primary diagnosis, from all public hospitals across Perth.

### Development of the traffic exposure metric

Traffic counts and geocoded count site locations were obtained from Main Roads Western Australia (MRWA) for the period 1984 to 2004. The MRWA site locations were selected because they represent actual or potential locations of traffic congestion, or where major traffic flows occur. The MRWA collects these data using automatic traffic recorders positioned at selected sites for 1 to 3 days' duration. These are supplemented with information from manual count surveys to obtain Annual Average Weekday Traffic (AAWT) counts, which is an estimated average 24-hour traffic volume for weekdays, adjusted for seasonal variations. Road categories were then defined using the MRWA Perth Metropolitan Functional Road Hierarchy [31], as follows: Primary Distributor (more than 15000 vehicles per day), District Distributor A (8000-15000 vehicles per day), District Distributor B (6000-8000 vehicles per day), Local Distributors (3000-6000 vehicles per day), and Access Roads (less than 3000 vehicles per day). A total of 423 count sites were used for the purposes of this project. Counts were adjusted by season and day of the week.

For this project, we also conducted a validation study using our own video monitoring at 18 MRWA sites, which showed that MRWA traffic counts were strongly correlated with those obtained via our video monitoring (r = 0.889).

### Assigning traffic exposures to cases and controls

**Figure 1:**
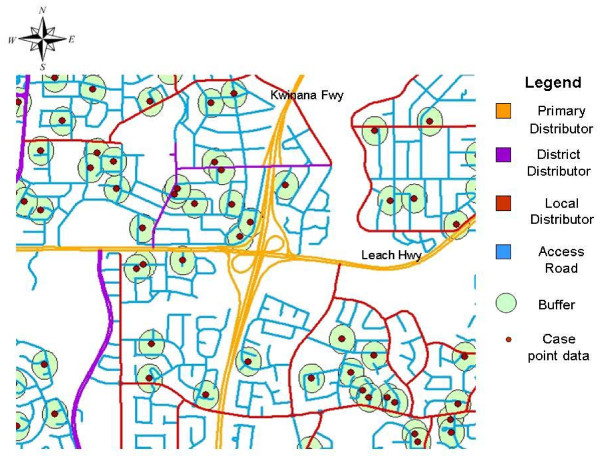
**Illustration of buffer zones used in traffic exposure metric**.

In brief, all cases and controls were mapped using the exact longitude and latitude coordinates of residential addresses. Circular buffer zones with n50 m, 100 m and 150 m radii were constructed around each subject using standard GIS software, ArcGIS (ESRI) (Figure 1). These buffer radii were chosen because previous studies have found the risk for respiratory symptoms was highest at distances up to 150 metres from major roads. Our spatial method assigned traffic weights to roads depending on their type (Primary Distributor, District Distributor A and B, Local Distributor and Access Road) based on the average daily vehicle counts as described above. The traffic exposure variable therefore used data on both traffic counts and type of road, and may be conceptualised as the total traffic count on the road segments contained within the circular buffer surrounding each subject's residential address.

### Statistical analysis

The total traffic counts within the buffer were log-transformed prior to regression analysis. Conditional logistic regression was used to calculate risk estimates for ED contact for asthma and the various traffic exposures at 50 m, 100 m and 150 m buffers. Estimates were adjusted for ethnicity and socio-economic status (SES). Ethnicity was dichotomised between Aboriginal and Torres Strait Islander subjects versus other ethnicities. Socioeconomic status was ascertained using the Socio-economic Index for Areas (SEIFA) Index of Relative Socio-Economic Disadvantage at the Collection District (CD) level. The SEIFA index corresponding to the CD of the subject's residential address was assigned to the subject. The CD is the smallest aggregate unit for which SEIFA was available. The SEIFA index is a validated and standardised metric that provides a comparative area-level measure on education, income, occupation, living conditions and access to services [32]. Statistical modelling was implemented using Stata MP10.

### Ethical issues

Ethical approval was obtained from The University of Western Australia Human Research Ethics Committee (RA/4/1/1511) and the Department of Health Western Australia Human Research Ethics Committee (former Confidentiality of Health Information Committee) (#200622).

## Results

### Demographics

A summary of case and control characteristics by the matching variables is shown in Table 1. Of the 434 subjects, the majority of the subjects were male (62%), in the 5-9 age group (48%) and presented at an ED in winter (32%). For the cases (that is, those with more than one ED contact in the specified duration), the minimum number of presentations was 2, maximum 13, mean = 3 (SD = 1.9) and median = 2. There were no significant differences in SEIFA scores between the case and control populations. Ethnicity data for the population included Aboriginal or Torres Strait Islander (ATSI) status, but no other classifications were available. A total of 15 (6.9%) cases and 13 (6.0%) controls were classified as Aboriginal or Torres Strait Islander (that is, 6.5% overall). No ethnicity information was recorded for 3 subjects (0.7%) across the study population overall, corresponding to 0% cases and 1.4% controls unclassified with respect to their ethnicity.

**Table 1:** Summary of case and control matching variables

	Cases	Controls	Total (%)
**All subjects**	217	217	434

**Gender**			
**Male**	134	134	268 (62)
**Female**	83	83	166 (38)

**Age strata**			
**0-4 years**	31	31	62 (14)
**5-9 years**	104	104	208 (48)
**10-14 years**	47	47	94 (22)
**15-19 years**	35	35	70 (16)

**Season**			
**Summer**	32	32	64 (15)
**Autumn**	58	58	116 (27)
**Winter**	70	70	140 (32)
**Spring**	57	57	114 (26)

### Traffic exposure metrics

**Figure 2:**
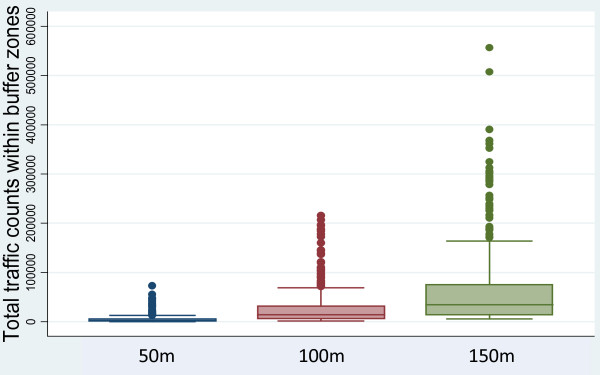
**Boxplots of untransformed traffic counts within 50 m, 100 m, and 150 m buffer zones**.

The summary statistics for the final traffic counts for each of the buffer zones are summarised in Table 2. The distributions of traffic counts are compared in Figure 2.

**Table 2:** Summary statistics of (untransformed) total traffic counts for each buffer size

	Mean/SD	Median	Minimum	Maximum	Range
**50 metre buffer**	4724 (8513)	1256	0	73337	73337

**100 metre buffer**	26882 (36859)	13701	1635	215768	214133

**150 metre buffer**	63183 (76772)	34121	5631	556184	550553

*SD = standard deviation

### Risk estimation for traffic exposure

Crude and adjusted odds ratios were calculated for three buffer zones at 50 metres, 100 meters and 150 metres (Table 3). For the crude odds ratios, increasing odds ratios were observed with increasing buffer sizes. For the adjusted estimates, only the 150 metre buffer yielded a statistically significant finding (OR = 1.24; 95% CI: 1.00-1.52). Minimal differences were observed between the crude and adjusted odds ratios for the exposure variable, suggesting that SEIFA and ethnicity were unlikely to be major confounders in this analysis. Regression diagnostics did not detect any major outliers, or values that displayed an unusual degree of influence or leverage. Sample sizes were too small to conduct stratified analysis by age, gender, ethnicity, season or socioeconomic status with a sufficient degree of precision.

**Table 3:** Odds ratios for log-transformed total traffic counts within 50 m, 100 m and 150 m buffer zones

	Crude odds ratio	95% CI	Adjusted odds ratio*	95% CI
**Log-unit increase in total traffic counts**				

**For 50 metre buffer**	1.07	0.91-1.26	1.07	0.91-1.25

**For 100 metre buffer**	1.21	1.00-1.46**	1.20	0.99-1.46

**For 150 metre buffer**	1.25	1.02-1.54**	1.24	1.00-1.52**

*Adjusted for SEIFA, ethnicity; **Significant at p ≤ 0.05

## Discussion

Our study revealed a statistically significant 24% increase in the risk of experiencing multiple emergency contacts for asthma for every log-unit of traffic exposure. This finding supports the conclusions of several studies showing an association between the frequency of health care contacts for asthma and proximity of the subject's residence to high-trafficked roads [15,16,19,33,34]. Our analysis also specifically examined the spatial relationship to roads at three different buffer sizes. Notably, the elevation in risk was only consistently observed using the 150 metre buffer to calculate the exposure metric. In contrast, no significant change in risk was observed for the 100 metre buffer for the adjusted estimates, although a significant 21% increase was noted for the crude estimates.

For the 50 metre buffer, no significant elevation in risk was detected for either the crude or adjusted estimates (unadjusted OR = 1.07; 95% CI: 0.91-1.26; adjusted OR = 1.07; 95% CI: 0.91-1.25). This pattern may be at least partially accounted for by the lack of discrimination of the exposure metric derived using the 50 metre buffer. The range of traffic counts at this size is narrow (as shown in Table 2 or graphically in Figure 2), which indicates that few cases or controls live in very close proximity to heavily trafficked sections. In contrast, as the buffer is expanded out to 100 metres and 150 metres, many more "segments" of road are included in the circular area. This translates to a marked increase in range of the assigned traffic counts, thereby equating to greater variations in exposure.

In this study, emergency department records were used as the source of asthma data. It has been noted that the use of reliable health service data, including those pertaining to emergency contacts, overcome the limitations of patient recall and reporting encountered in other study designs [27]. One possible constraint is that information was retrieved only from hospitals with public emergency departments within Perth, and thus any information from private hospital emergency departments was not available. However, given that both the cases and controls were derived from that same source (that is, emergency departments in Perth public hospitals), any confounding effects of patients who access public versus public hospitals - such as differential socioeconomic status - would tend to have been minimised. Access to public hospitals is free in Australia, thereby reducing the barrier of cost.

Because of the reliance on de-identified medical records in this study, there were a limited number of potential confounders obtainable for analysis: sex, age, socio-economic status, and ethnicity. (Season was also partially adjusted for, as described in the Methods.) Individual data on other important exposures - including smoking status of the child or parent, family history of asthma, contact with indoor air pollutants and allergens - were not available. Another significant limitation is the failure to account for individual's spatio-temporal activity patterns. This study relies on the assumption that most of the relevant exposure to air pollutants for individuals are traffic related and are from residential location, rather than from other locations such as day-care centres, schools, recreational areas, work, or through commuting [35,36]. The geographical information used for the exposure assessment was the home address when presented at the emergency department, and is therefore is restricted to particular points in time.

Other urban pollutants apart from traffic emissions may account for some of the exposure. In Perth, wood is the main solid fuel for home heaters, and domestic wood combustion contributes significantly to levels of particulate matter, particularly during the winter [37]. Emissions from industry are possible, although the predominant sources are located to the east of the study area, and given the prevailing winds are unlikely to be a major contributor to air quality relative to vehicle emissions. However, these risk factors do not explain the effects because they are unlikely to be related to traffic.

No meteorological data were included in the analysis and there were no monitoring stations within the study area from which to obtain historical data. Previous studies have indicated that the dispersion of air pollutants from a major vehicle corridor is not uniform and may be affected by meteorological conditions. Concentrations of air pollutants, such as NO_2 _and soot, decrease with increased distance from a motorway [38,39], but dispersion can be affected significantly by wind directions and patterns [40,41].

Surprisingly, our study did not provide evidence of relationship between the frequency of emergency department contacts for asthma and socio-economic status (SES). This conflicts with a study by Eagan et al [42] that showed lower SES (based on educational level) to be strongly associated with asthma and respiratory symptoms. In a longitudinal study, Hedlund [43] also found that low SES was a risk factor or the development of asthma, symptoms common in asthma and chronic productive cough. In our analysis, aggregated area data (that is, based on CDs) were used to assign socioeconomic status as opposed to individual estimates, and there is the possibility of ecological bias arising from the assumption of individual-level socioeconomic status from area-level classification.

## Conclusions

Our study revealed a significant 24% increase in the risk of experiencing multiple emergency department contacts for asthma for every log-unit of traffic exposure. The findings of this study provide support for the hypothesis that traffic-related air pollution is potentially hazardous to children with asthma, and proximity to busy roadways tends to increase the risk for emergency presentations. This study used advanced GIS software to establish buffer zones around a geocoded residential location to provide a more accurate assessment of exposure to traffic emissions compared to existing spatial methods, thereby providing a quantification of the ranges over which pollutants may exert a health effect.

Although there were some limitations in this approach, there is future potential for estimates from the metric to be integrated with personal-level information to provide a more complete exposure profile. It is anticipated that the traffic count metric presented would have a number of applications in other urban contexts and could be extended to other disease outcomes relating to traffic emissions, such as cardiovascular disease and adverse birth outcomes.

## List of abbreviations

AAWT: Annual Average Weekday Traffic; ATSI: Aboriginal or Torres Strait Islander; CD: census district; CI: confidence interval; CO: carbon monoxide; CRC: Cooperative Research Centre; ED: Emergency Department; EDIS: Emergency Department Information System; GIS: Geographical Information Systems; MRWA: Main Roads Western Australia; NOx: oxides of nitrogen; OR: odds ratio; PM: particulate matter; SEIFA: Socio-economic Index for Areas; SES: socio-economic status; SOx: oxides of sulphur; VOCs: volatile organic compounds;

## Competing interests

The authors declare that they have no competing interests.

## Authors' contributions

AC conceived of the study, developed the epidemiological design and drafted the manuscript. JAMdeV developed the GIS models and assisted with data analysis. GP participated in the design of the study and performed the statistical analysis. AJ participated in the design of the study and assisted with interpretation. PW participated in the development of the study, and participated in its design and coordination and helped to draft the manuscript. All authors read and approved the final manuscript.

## Acknowledgements

The authors would like to acknowledge CRC Asthma and Airways for financial support of the work described herein. We would also like to thank Andrew Wu for his assistance in developing the GIS model.
